# Chloride binding and mobility in sodium carbonate-activated slag pastes and mortars

**DOI:** 10.1617/s11527-017-1121-8

**Published:** 2017-12-01

**Authors:** Xinyuan Ke, Susan A. Bernal, Oday H. Hussein, John L. Provis

**Affiliations:** Department of Materials Science and Engineering, Sir Robert Hadfield Building, The University of Sheffield, Sheffield, S1 3JD UK

**Keywords:** Sodium carbonate-activated slag, Layered double hydroxide, Chloride, Durability, Microstructure

## Abstract

This study evaluates the chloride binding capacity and the migration of chloride in sodium carbonate-activated slag cements and mortars. The effect on chloride mobility and binding of adding a calcined layered double hydroxide (CLDH) to the binder mix was also assessed. Significantly improved durability characteristics can be achieved for sodium carbonate-activated slag mortars by the addition of small fractions of CLDH, as a consequence of a higher degree of reaction, higher chloride binding capacity, and the refined pore structures present in these modified materials, in comparison with alkali-activated cements produced without CLDH. The addition of CLDH enables the production of sodium carbonate-activated slag cements with notably reduced chloride ingress compared to silicate activated slag cements.

## Introduction

Alkali-activated materials have recently gained significant interest as an alternative to Portland cement in many applications [1–3]. Although alkali-activated slag (AAS) cements can be produced with much lower global warming potentials than Portland cement (PC) [4], the use of sodium hydroxide and sodium silicate as activators can bring higher impacts than that of PC in other environmental aspects, including human toxicity, fresh water and marine ecotoxicity [4–6]. In the search for more cost-effective, low-toxicity and environmentally friendly alternatives, the use of near-neutral salts such as sodium carbonate as activators for blast furnace slag has attracted the attention of academia and industry [7–14]. However, the factors controlling the durability and mechanical performance of sodium carbonate-activated slag cement are not yet fully understood. The phase assemblages of AAS cements are mainly controlled by the chemistry of the slags and activators used [7, 15–18], and the microstructures and mechanical properties of AAS cements are also strongly influenced by the type of activator [19–22].

The durability performance of AAS cements, as is the case for materials based on Portland cement, is closely related to the microstructural features of the binder, as well as the chemical reactions that can take place between the binder reaction products and the chemical species present in the service environment. Free chloride in a cement pore solution increases the likelihood of corrosion of steel reinforcement, and reduces the durability of concrete structures [23–25]. Measurement of the ionic transport of free chloride in concrete structures is therefore particularly important for service life prediction [26, 27]. The diffusivity of chloride in a cementitious binder is often evaluated by measuring its diffusion and/or migration coefficients.

The interactions of chloride with saturated cementitious materials are governed by physico-chemical phenomena associated with its diffusion through the pore network of hardened concrete, and its tendency to chemically bind to the hydrated phases present. The chemistry of the aqueous environment (e.g. pH, ionic concentration), and the phase assemblage of the cementitious matrix, must be taken into account when studying the chloride binding capacities of AAS cements [25, 28, 29]. The aqueous environment under which the chloride binding capacity is determined in laboratory tests must therefore be comparable to that of the pore solution chemistry of the hardened cement, so that the results are sufficiently representative. Nonetheless, chloride binding capacities of cementitious materials are normally measured in chloride solutions at near-neutral pH [29, 30]. This may lead to overestimation of the chloride binding capacity, as a very high initial [Cl^−^]/[OH^−^] ratio will favour the binding of chlorides in the solid phases, either through ion exchange or surface adsorption [31]. The use of a highly alkaline chloride-rich simulated pore solution mitigates this effect.

In our previous work, calcined layered double hydroxide (CLDH) was incorporated in sodium carbonate-activated slag paste for improved control of the setting time [14]. CLDH is produced by thermal treatment of a layered double hydroxide mineral such as hydrotalcite, which contains a positively charged layer structure that allows exchange of interlayer anions [32–34]. Recently, CLDH has begun to be used as a ‘smart’ chemical addition for cementitious materials due to its ion-exchange properties, enhancing performance and durability [14, 35–38]. A recent study also demonstrated the high chloride binding capacity of the different layered double hydroxide (LDH) type phases typically identified in alkali-activated slag paste [39]. Although LDHs can chemically bind chloride, the potential role of this behaviour in determining the durability of AAS cement is yet to be fully elucidated.

In this study, chemical binding capacities of AAS cements were determined using simulated chloride-rich pore solutions. Sodium carbonate-activated slag pastes with 0 and 5 wt% CLDH addition were studied, and a sodium silicate-activated slag paste was tested as a reference sample. Chloride binding isotherms of all samples in the simulated pore solutions were calculated. The changes in phase assemblage after exposure to chloride-rich simulated pore solutions have been characterised using X-ray diffraction (XRD) and scanning electron microscopy (SEM). Mortar specimens with equivalent compositions to the sodium carbonate and sodium silicate-activated slag pastes were prepared, and tested according to Nordtest NT Build 492 for chloride migration as a function of curing time and mix design. Compressive strengths of mortars at different curing durations, and pore size distributions of selected samples according to mercury intrusion porosimetry (MIP), were also determined. The chloride binding isotherms determined for paste samples can then be used in assessing the factors influencing the migration coefficients measured by accelerated testing of the mortars.

## Experimental methods

### Materials

A commercial blast furnace slag was used in this study, with a chemical composition of 41.3 wt% CaO, 36.0 wt% SiO_2_, 11.3 wt% Al_2_O_3_, and 6.5 wt% MgO; other oxides sum to 2.5 wt%, and loss on ignition at 1000 °C was 2.0 wt%. This slag had a Blaine fineness of 5056 ± 22 cm^2^/g, and a d_50_ of 11.2 μm was determined by laser diffraction particle size analysis.

Analytical grade anhydrous sodium silicate powder (Sigma Aldrich, Na_2_SiO_3_ ≥ 99.5%), with a SiO_2_/Na_2_O molar ratio of 1, and sodium carbonate powder (Sigma Aldrich, Na_2_CO_3_ ≥ 99.5%) were dissolved into water to prepare the activator solutions used in this study. The CLDH used in this study was prepared using the same procedure as in previous studies [14]. CEN Standard Sand, certified in accordance with EN 196-1:2005, was used in all mortar samples.

### Sample preparation and test methods

#### Alkali-activated slag pastes and mortars

The alkali-activated slag pastes and mortars were prepared according to the mix designs shown in Table 1. Three types of slag pastes were prepared: slag paste produced with sodium carbonate activator without CLDH (denoted P-NC-0) and with CLDH addition (P-NC-1), or sodium silicate activator without CLDH addition (P-NS-0). For mix design purposes the CLDH added to these cements was considered as an additive, and the amount of activator and water added to each unit mass of slag was kept constant. A previous study by the authors [14] demonstrates that CLDH rehydration will consume a fraction of the water added into these cements; however, the changes in the overall water/binder ratio are negligible at 5 wt% CLDH addition. The pastes were cured for 28 days before being crushed for chloride binding experiments. Correspondingly, three types of mortars were prepared: sodium carbonate-activated mortars without CLDH (M-NC-0) and with CLDH addition (M-NC-1), and sodium silicate-activated mortars without CLDH addition (M-NS-0). All samples were cured in tightly sealed plastic bags for up to 180 days.

**Table 1:** Mix design of the paste (P) and mortar (M) samples assessed in this study (for each of 100 g anhydrous slag used)

Sample ID	Activator type	Mass of slag (g)	Mass of the activator (g)	CLDH (g)	Water (g)	Sand (g)	^+^w/b
P-NC-0	Na_2_CO_3_	100	8	0	43.2	0	0.4
P-NC-1		100	8	5	43.2	0	0.4
P-NS-0	Na_2_SiO_3_	100	7	0	42.8	0	0.4
M-NC-0	Na_2_CO_3_	100	8	0	43.2	300	0.4
M-NC-1		100	8	5	43.2	300	0.4
M-NS-0	Na_2_SiO_3_	100	7	0	42.8	300	0.4

^+^w/b = water/binder mass ratio (where binder is defined as mass of slag plus activator solids)

#### Chloride binding capacity of AAS pastes

Four chloride-rich simulated pore solutions (denoted CH-1 to CH-4) were prepared using a mixture of sodium hydroxide (Sigma Aldrich, NaOH ≥ 98.0%) and NaCl (Sigma Aldrich, NaCl ≥ 99.5%), keeping the total Na^+^ concentration constant at 1.0 mol/L, but with [Cl^−^]/[OH^−^] ratios of 0.1, 0.3, 1.0 and 3.0 respectively.

After 28 days of curing, each type of slag paste prepared in this study was crushed with a hammer in sealed plastic bags, and sieved to obtain samples with particle sizes ranging from 0.25 to 0.6 mm. Immediately after crushing, the sieved paste powders were added to chloride-rich solutions with a solid/liquid ratio of 1:7 (2 g solids to 14 g solution) in 15 mL centrifuge tubes. The tubes were sealed with Parafilm, and stored horizontally at 23 ± 2 °C for 2 months to allow them to reach reaction equilibrium, agitated in a roller mixer for 1 h once per week.

After 2 months, the samples were separated using a centrifuge (Heraeus Biofuge Primo, 4000 rpm for 6 min). The supernatants were collected to enable calculation of chloride binding capacity. The chloride concentration and pH in the supernatants were measured using a chloride ion-selective electrode (Cole-Parmer Epoxy solid-state chloride electrode, accuracy ± 2%) and pH meter (Oakton Acorn Series). The chloride binding capacity of each slag paste in each aqueous solutions was calculated using Eq. (1).

1 $$Q_{e} = (C_{e} - C_{0} ) \cdot V/m_{\text{input}}$$

*Q*_*e*_ Chloride binding capacity of solid, mg/g (by dry mass of initial solid). *C*_*e*_ Chloride concentration of the supernatant solution, mol/L. *C*_0_ Initial chloride concentration, mol/L. *V* Volume of solution, mL. *m*_input_ Initial mass of solid, g.

The remaining solids separated from chloride-rich simulated pore solutions were washed using Milli-Q water following the RILEM recommendations for analysis of water soluble chloride content in concrete [40], and then dried in a desiccator with controlled relative humidity at 30 ± 3% (reached using saturated CaCl_2_ salt) for 4 days prior to further analysis. Samples were pulverised and analysed via XRD. Additionally, for the pastes exposed to the solution CH-3, part of the paste specimen was embedded in epoxy resin and polished for SEM–EDX analysis, and part was pulverised and analysed by thermogravimetry coupled with mass spectrometry (TG–MS).

#### Test methods for AAS mortars

Mercury intrusion porosity (MIP) was used according to the test procedure recommended by Ma [41]. Samples were sectioned from cubic specimens using a slow saw, with dimensions of no less than 5 mm each side. About 3 g of mortar samples were used in each measurement to ensure representative results. The sectioned mortar samples were immersed in isopropanol for 24 h, followed by vacuum drying for 3 days for complete removal of pore water. The MIP tests were then conducted using a Micromeritics Autopore 9600 Mercury Porosimeter, assuming an intrusion contact angle of 130° and an extrusion contact angle of 104°.

Mortar cubes with dimensions of 50 × 50 × 50 mm were used to test compressive strength, using an automatic compressive testing instrument (Controls Automax5), with a loading speed of 0.25 MPa/s. Triplicate samples were measured per formulation per curing age.

Non-steady state chloride migration coefficients of mortars were determined following the NordTest method NT BUILD 492 [42]. Mortar discs (∅100 × 50 mm) were used, and duplicate samples were prepared per formulation per curing age. Prior to the accelerated chloride migration test, each of the discs was immersed in 1.0 M NaOH solution under vacuum to obtain pore water-saturated samples; the immersion fluid was selected to have a comparable (but simplified) composition to those predicted by thermodynamic modelling for the cements assessed [43]. At the end of the test, the disc sample was split, and immediately sprayed with 0.1 M silver nitrate (AgNO_3_) to obtain the chloride ingress profile [44]. The non-steady-state migration coefficient was then calculated based on [42], but with a modified value of 0.16 N selected as the chloride concentration at which the color changes, due to the high alkalinity in the pore solution [45].

## Results and discussion

### Chloride binding capacity of alkali-activated slag pastes

**Fig. 1:**
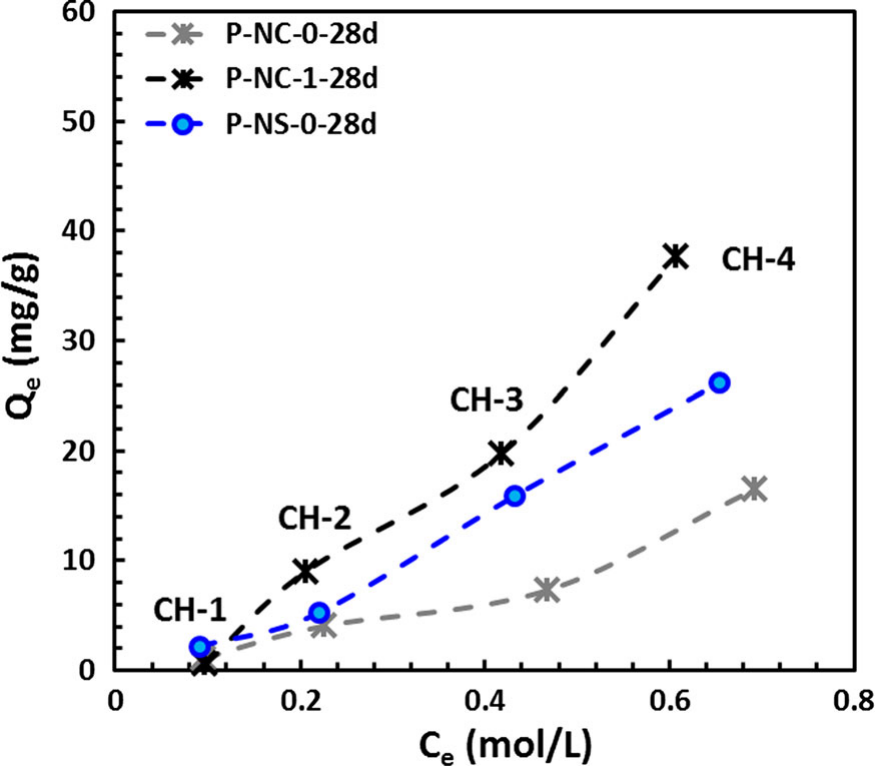
Chloride binding capacities of 28-day cured alkali-activated slag pastes determined in chloride-rich simulated pore solutions

For the specimens assessed, the chloride binding capacity (*Q*_*e*_) of each slag paste under various aqueous environments was calculated using Eq. (1). Figure 1 shows the chloride binding capacities calculated, as a function of the [Cl^−^/OH^−^] ratio in the exposure solution. The same trend was identified in all three samples, where the binding increases at higher [Cl^−^/OH^−^] ratios. The reduced binding capacity at a low [Cl^−^/OH^−^] ratio (CH-1) could be a consequence of the low chloride concentration, and the competition between hydroxyl ions and chloride for potential anion binding sites in the hydrotalcite type phases forming in these cements [39]. Among the three samples assessed, the chloride binding capacity of sodium carbonate-activated slag paste is much lower than that of sodium silicate-activated slag paste, while the chloride binding capacity of the paste containing CLDH (P-NC-1) is higher than that of samples without this addition, independent of the activator used.

Both hydrotalcite-group phases and carbonate-AFm phases (monocarbonate or hemicarbonate) are considered to be effective chloride binding phases [39, 46]. The main reaction product in AAS cements, which is a sodium–aluminium substituted calcium silicate hydrate (C–(N)–A–S–H) type gel, can also bind chlorides, but with a much lower binding capacity than LDHs [47]. Between the P-NC-0 and P-NS-0 samples, the P-NC-0 sample might contain less binder materials as a result of slower development of reaction comparing with the sodium silicate-activated samples [9, 10]. Also, the hydrotalcite-like phase in the sodium carbonate-activated samples might contain some carbonate anions [14], leaving less sites available for binding of Cl^−^ [39]. As for CLDH modified samples, the incorporation of CLDH in sodium carbonate-activated slag cements increases the content of hydrotalcite-like phases in the binder. Although the content of AFm phases would decrease [14], the overall content of LDHs (hydrotalcite-like phase + AFm phases) will still be higher than in AAS paste without CLDH modification. Thus, the competition between CO_3_^2−^ and Cl^−^ for binding sites in hydrotalcite-like phase would be less significant as the abundance of the hydrotalcite-like phase is high. Also, a higher degree of reaction has been observed in CLDH modified sodium carbonate-activated slag paste, indicating that there are more reaction products available to potentially bind chloride [14]. The increased chloride binding capacity in CLDH modified AAS pastes is therefore a consequence of the higher overall LDH content, compared with non-modified cements. However, to better understand the changes in the mineralogy of AAS binders after exposure to external chlorides, it is essential to study the solid samples after immersion in chloride-rich simulated pore solutions.

### Mineralogy of alkali-activated slag pastes after exposure to chloride-rich solutions

#### X-ray diffraction (XRD)

**Fig. 2:**
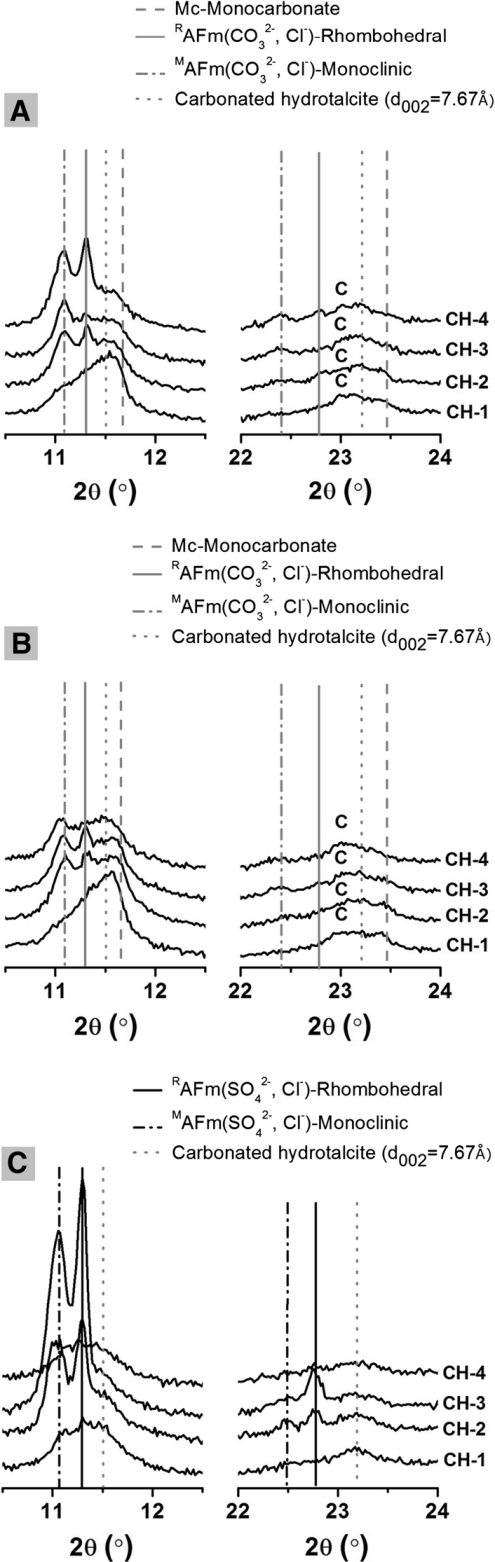
X-ray diffraction patterns of 28-day cured sodium carbonate-activated slag pastes **a** without CLDH (P-NC-0), and **b** with CLDH (P-NC-1) addition; and **c** sodium silicate-activated slag paste (P-NS-0), at chloride binding equilibrium in different simulated pore solutions. C indicates a reflection due to calcite (PDF# 00-005-0586)

Figure 2 shows the XRD patterns in selected angle ranges to highlight the changes in the reflections of LDH phases upon chloride uptake. In both sodium carbonate-activated samples, with or without CLDH addition, the intensity of the main reflection peak assigned to the AFm-structured monocarbonate phase decreases as the chloride binding increases. Two polymorphs of Friedel’s salt are observed in these specimens, ^R^AFm-(CO_3_^2−^, Cl^−^) which is close to the structure of rhombohedral hydrocalumite (Ca_2_Al(OH)_6_Cl·2H_2_O, powder diffraction file (PDF) #00-035-0105), and ^M^AFm-(CO_3_^2−^, Cl^−^) which is close to monoclinic hydrocalumite (Ca_2_Al(OH)_6_Cl·2H_2_O, PDF# 00-019-0202), but has a lower basal peak position [48]. The hydrocalumite-type phases formed in sodium carbonate-activated samples were transformed from monocarbonate to Friedel’s salt type phases, most likely by replacement of some of the interlayer CO_3_^2−^ ions with Cl^−^ ions, as has been proposed by Mesbah et al. [46, 48] The transformation between these two polymorphs of Friedel’s salt is described in the literature to be mainly temperature controlled, with the rhombohedral structure preferred at higher temperature (above 35 °C) [46, 49, 50]. The differences in interlayer species might affect the transition temperature, however, there has not been any evidence directly correlating the transition between the two polymorphs with changes in interlayer chloride content.

The main reflection peak of a CO_2_-containing hydrotalcite-like phase with basal spacing 7.67 Å is shown in Fig. 2, as a guideline for identifying hydrotalcite-like phases. In sodium carbonate activated slag pastes (P-NC-0) the interlayer species in the hydrotalcite-like phase can be a mixture of Cl^−^, OH^−^ and CO_3_^2−^, considering the chemical composition of the aqueous phase at equilibrium before separation. The intensities of reflections assigned to AFm-(CO_3_, Cl) are much higher in samples without CLDH (Fig. 2b) than in CLDH-containing specimens (Fig. 2a). Less AFm phase was formed in sodium carbonate-activated slag pastes with added CLDH (P-NC-1), as observed in [14], reducing the amount of monocarbonate available to chemically bind chlorides. This emphasises the role of CLDH in increasing the chloride binding capacity of AAS cements, as its inclusion modifies the phase assemblage of these cements, impacting how chloride binding occurs.

In sodium silicate-activated samples (P-NS-0), the poorly crystalline AFm phase (strätlingite-like) transformed into a Friedel’s salt-like phase after exposure to a chloride-rich solution. Two polymorphs of chloro-carboaluminate phases were again identified after chloride binding in this specimen. The intensities of the basal peaks assigned to both phases increase as the external [Cl^−^]/[OH^−^] ratio rises. This is associated with an increased formation of chloride-bearing AFm phases. However, in specimens exposed to solutions with the highest [Cl^−^]/[OH^−^] ratio (CH-4), the peaks corresponding to chloride bearing AFm phases were not clearly identifiable. As discussed in a previous study [39], any Friedel’s salt-like phases formed through the uptake of chlorides by strätlingite will decompose even in alkaline solution (pH around 13.6) in the presence of carbonate ions. It is possible that according to such a mechanism, a Friedel’s salt-like phase was originally formed in this paste, but decomposed during sample processing or analysis.

#### Scanning electron microscopy (SEM–EDX)

**Fig. 3:**
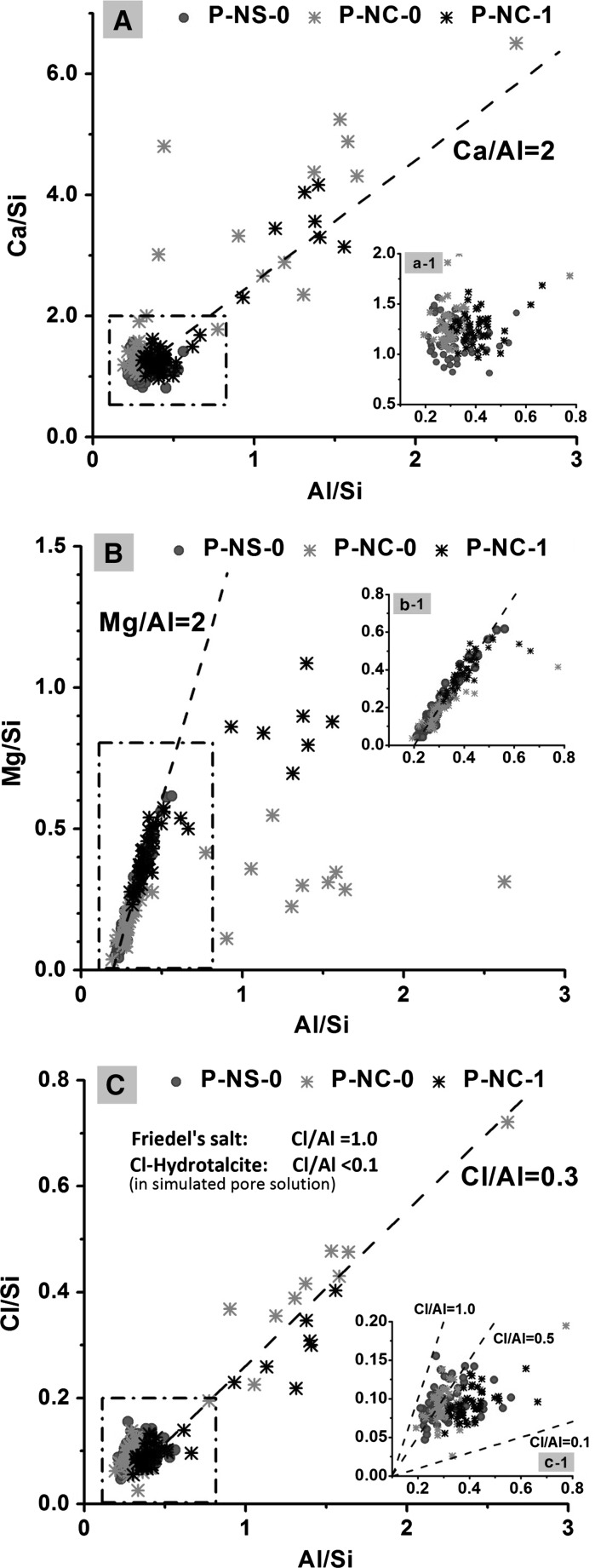
Atomic ratios calculated from EDX data for 28-day cured alkali-activated slag pastes after exposure to solution CH-3: **a** and (a-1) plotted as Ca/Si versus Al/Si, **b** and (b-1) plotted as Mg/Si versus Al/Si, **c** and (c-1) plotted as Cl/Si versus Al/Si

Figure 3 shows the atomic ratios calculated from EDX data for AAS pastes embedded in epoxy resin, after exposure to the chloride-rich solution CH-3. The dashed lines in Fig. 3a, b, showing the ratios Ca/Al = 2 and Mg/Al = 2 respectively, are included to aid identification of the composition regions where AFm and/or hydrotalcite-like phases have been reported [51]. The dashed lines of different Cl/Al ratios in Fig. 3c are given as a guide in evaluating the Cl content of the reaction products. The maximum Cl/Al ratio possible in an AFm phase is around 1.0, as seen in Friedel’s salt [52], while the highest Cl/Al ratio in a hydrotalcite-like phase measured in simulated pore solutions was around 0.1, as calculated in [39].

The EDX plots for chloride-bearing sodium carbonate-activated samples, shown in Fig. 3a, b, are generally similar to those of sodium carbonate-activated samples without exposure to chlorides [14]. The data points in Fig. 3a with Al/Si ratios higher than 0.8 and Ca/Si ratios around Ca/Al = 2 suggest the formation of crystallised AFm phases in both of the sodium carbonate-activated slag pastes, with and without inclusion of CLDH (P-NC-0 and P-NC-1). Between these two samples, the Mg/Si atomic ratio in sample P-NC-1 is higher than that in P-NC-0 (Fig. 3b), consistent with the addition of the CLDH. These results also indicate the existence of the hydrotalcite-like phase intermixed with crystallised AFm phases in sample P-NC-1 [14]. However, the Cl/Al ratios in data points collected from regions mainly consisting of hydrotalcite-like and AFm (Al/Si > 0.8) phases seem to be similar between sample P-NC-0-28d and P-NC-1 (Fig. 3c). It seems that the bulk Cl/Al ratios in the Friedel’s salt-like AFm-(CO_3_^2−^, Cl^−^) phase and the chloride-bearing hydrotalcite-like phase are similar, at around Cl/Al = 0.3.

Compositions corresponding to a discrete AFm phase were not observed in EDX analysis of sample P-NS-0 after exposure to solution CH-3, even though a Friedel’s salt-like phase was identified though XRD (Fig. 2). This could possibly be explained by the fact that the AFm phase in sodium silicate-activated slag paste is intimately intermixed with C–(N)–A–S–H gel [53, 54], and therefore it is not distinguishable by SEM–EDX analysis.

The inset plots in Fig. 3 show an expanded view of the data points with Al/Si ratios between 0.1 and 0.8, representing data collected from locations where the C–(N)–A–S–H type gel is the dominant phase, most likely from the outer product with intermixed hydrotalcite-like and AFm phases [55–57]. From the inset plot in Fig. 3a, the Ca/Si ratios in sodium carbonate-activated samples with and without CLDH addition are similar to each other, and slightly higher than in sodium silicate activated slag paste, in accordance with the observations in previous study without chloride exposure [14]. Negligible differences between these three samples were observed from the inset plot in Fig. 3b. Comparing with sample P-NC-0, the overall Al/Si ratio is lower in P-NS-0 and higher in P-NC-1, due to an additional supply of Si from the activator (sodium silicate) and Al from the added CLDH, respectively. In the Fig. 3c inset plot, the sample P-NS-0 showed more EDX points distributed in regions with higher Cl/Al ratios, comparing with both sodium carbonate-activated samples. The higher Cl/Al ratio in P-NS-0 sample is mostly attributed to the intermixed Friedel’s salt-like phase, as observed from XRD patterns (Fig. 2c). Part of the Al in the bulk chemical composition is present in the C–(A)–S–H type gel, and the highest possible Al/Si ratio in the C–(N)–A–S–H type gel (single phase) is limited to between 0.1 to 0.167 by its cross-linked structure [58, 59], therefore the actual Cl/Al ratios in the Friedel’s salt-like phase could be close to 1.0 (or mostly within the Cl/Al region between 1.0 to 0.5). As for the two samples activated using sodium carbonate, it might seem that sample P-NC-0 has a higher Cl/Al ratio than P-NC-1; however, this could be attributed to a higher Al content in P-NC-1 (due to the addition of CLDH) rather than a higher Cl content in P-NC-0. Differences in Ca/Si and Al/Si ratios of C–(N)–A–S–H type gels would be expected to lead to different chloride binding capacities [47]. However, as shown in Fig. 3a, b, the bulk Ca/Si and Al/Si ratios of the C–(N)–A–S–H gels formed in all three samples appeared to be quite similar, although the high intermixing with the LDH phases means that it is difficult to clearly distinguish chlorides interacting specifically with the C–(N)–A–S–H type phases by EDX.

### Other factors related to mobility of chlorides in alkali-activated slag mortars

#### Pore structure (MIP)

For cementitious materials, mercury porosimetry can effectively measure the volume of pores that are directly connected to the sample surface, or connected through large pores [60]. In both paste and mortar samples, the distribution of overall intrudable porosity measured using MIP can reflect the range of pore entry sizes present, which is directly related to permeability and thus ionic transport properties in cementitious materials.

**Fig. 4:**
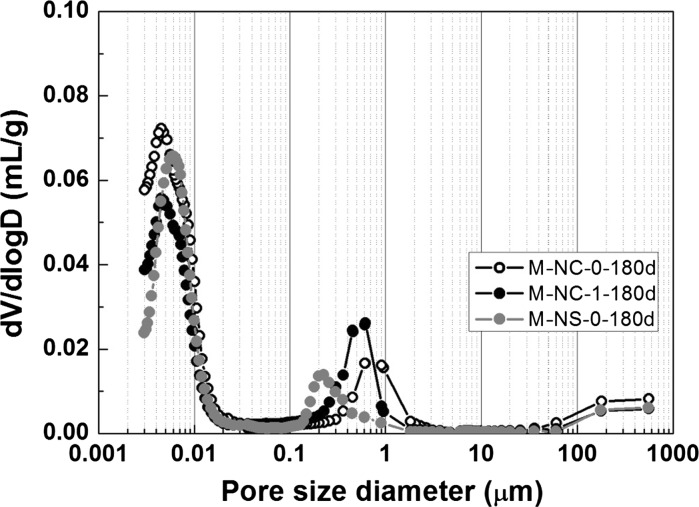
Differential pore volume distributions of mortar samples NC-0, NC-1, and NS-0 at 180 days of curing

Figure 4 shows the pore size distributions of mortar samples after 180 days of curing as measured by MIP. Sample M-NC-0-180d has the highest overall intrudable porosity, while sample M-NS-0-180d has the lowest. The CLDH-modified sample, M-NC-1-180d, showed much lower intrudable porosity than the unmodified sample M-NC-0-180d, and only slightly higher than that of M-NS-0-180d. Table 2 shows the fraction of gel pores (< 10 nm) [61] within the total intrudable porosities. Based on the results reported in Fig. 4 and Table 2, it is observed that the lower overall intrudable porosity of the CLDH modified sample (M-NC-1-180d) than the unmodified sample (M-NC-0-180d) is mainly due to the existence of less gel pores. The absolute value of permeable porosity (> 10 nm) of these two samples is almost the same. Compared with sodium silicate-activated mortars, sodium carbonate-activated mortars exhibit a higher overall intrudable porosity as well as a higher percentage of gel pores, while CLDH-modified sodium carbonate-activated mortar has a lower percentage of gel pores despite its slightly higher overall intrudable porosity.

**Table 2:** Summary of intrudable porosities, critical pore diameters and apparent bulk densities of mortar samples

	Intrudable porosity (%)				
	Total	Gel pores (< 0.01 μm)	Permeable pores (> 0.01 μm)	Critical pore diameter (μm)	Bulk density (g/mL)
M-NC-0-180d	11.94	6.98	4.96	0.763	2.2
M-NC-1-180d	9.93	5.18	4.75	0.532	2.3
M-NS-0-180d	9.37	5.95	3.42	0.209	2.3

Previous work showed that CLDH incorporated in sodium carbonate-activated slag paste consumes free water and increases the degree of reaction of slag, as CLDH particles act as nucleation seeding points [14]. Between sample M-NC-0-180d and M-NC-1-180d, the addition of CLDH slightly reduced the overall water/solids ratio, increased the degree of reaction in sodium carbonate-activated slag cement at this given curing time, and at the same time performs partially as a filler [14], all of which factors contribute to the lower critical pore diameter observed in sample M-NC-180d [62]. The more homogeneous microstructure in the paste section (between the unreacted slag grains) in CLDH-modified sodium carbonate-activated slag paste also contributes to the lower overall porosity of mortars produced with these additions.

#### Compressive strength

**Fig. 5:**
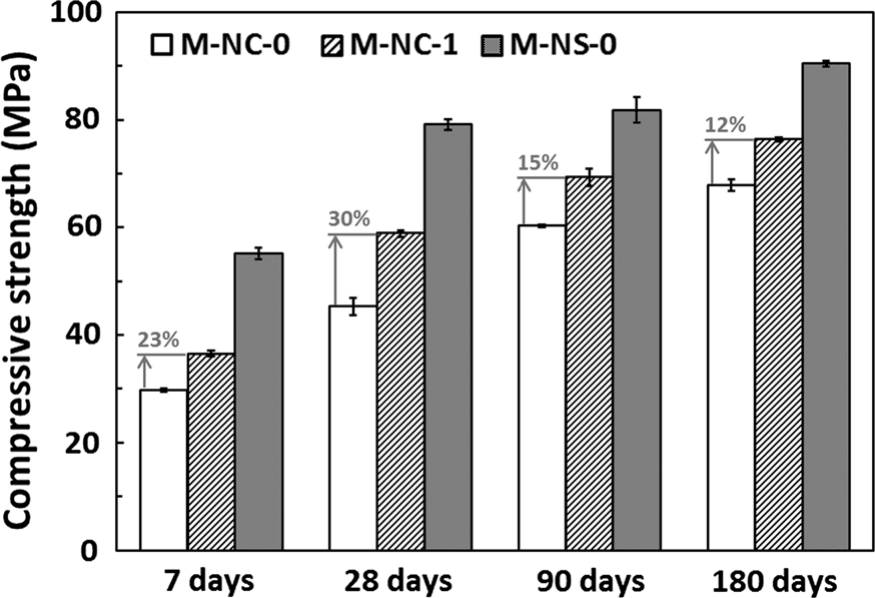
Compressive strength of M-NC-0, M-NC-1, and M-NS-0 mortar cubes at 7, 28, 90, and 180 days of curing. The results displayed are the mean and standard deviation of three replicates

Figure 5 shows that at each of the curing ages tested here, between the three alkali-activated materials studied, the compressive strength was consistently higher in M-NS-0 than M-NC-1, which was in turn stronger than M-NC-0. The addition of 5 wt% CLDH to sodium carbonate-activated slag mortar promoted a significantly increased compressive strength, compared with that obtained for sodium carbonate-activated slag mortars without CLDH, by at least 12%.

The lower strength development of sodium carbonate activated slag cement in comparison with sodium silicate-activated slag cement (under otherwise similar formulation conditions) has been reported in the literature [17, 19, 20, 63], and is associated with the differences in phase assemblage and permeability developed in activated slag systems when using different activators. A higher overall volume of connected (intrudable) pores would be expected to lead to lower compressive strength [19], and the correlation between these parameters observed here at 180 days (Figs. 4 and 5) is consistent with such a relationship.

Also, it is worthwhile to note from the literature that, when CLDH was blended with calcium sulfoaluminate cement, changes in sample strength were insignificant [35]; when it was blended into Portland cement, decreases in sample strength were observed [36, 37]. In those cementitious systems the hydrotalcite-like phase is not an intrinsic reaction product; therefore the recrystallised CLDH in those systems performs most likely just as a filler, even though it may also consume water as it rehydrates, and reduce the overall water/binder ratio in those systems. In alkali-activated slag systems the higher compressive strength identified in CLDH modified samples is a direct consequence of the lower porosity and higher degree of reaction of these binders, demonstrating the unique benefit of CLDH in tailoring sodium carbonate-activated slag cement.

#### Non-steady state chloride migration coefficient (NT BUILD 492)

**Fig. 6:**
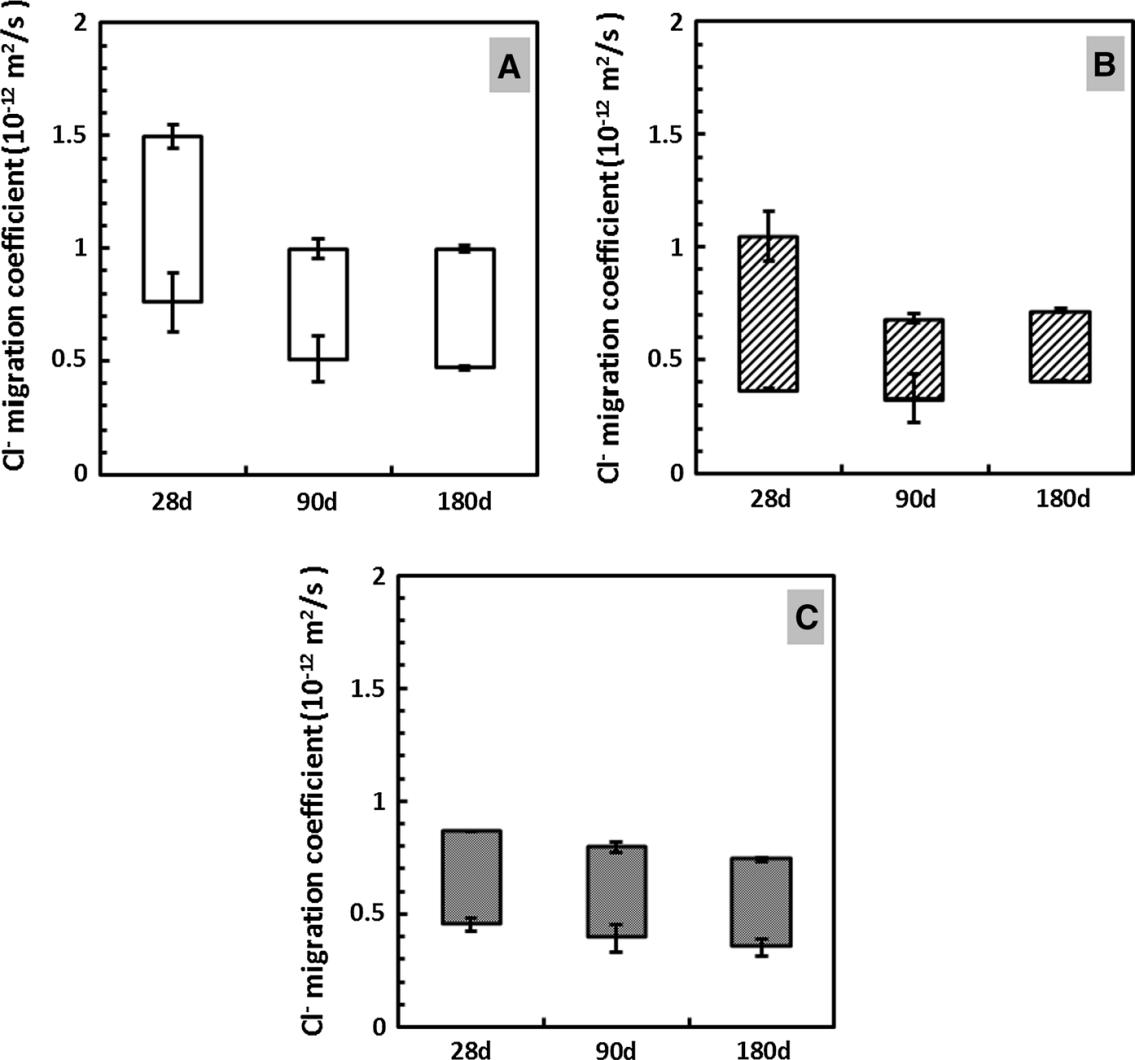
Chloride migration coefficient (according to application of NT BUILD 492 with calculation method modified as described in Sect. 2) of **a** M-NC-0, **b** M-NC-1, and **c** M-NS-0 mortar, after 28, 90, and 180 days of curing. The results displayed are the mean and standard deviation calculated from the highest (upper limit) and lowest (lower limit) chloride ingress depths among 7 readings on each of duplicate specimens

The NordTest accelerated migration test results for the three mortar mixes assessed are reported in Fig. 6. The lowest and highest possible chloride migration coefficients of these samples have been calculated based on the maximum and minimum points of chloride ingress into the samples tested, to give an illustration of the within-sample variability inherent in this test. This is important because in chloride attack on reinforced concrete, it is the first point of failure (i.e. the maximum depth of ingress) that will determine the service life of an element by inducing corrosion, and so this is more important than simply determining the average depth of ingress over an entire sample.

For sodium carbonate-activated samples, shown in Fig. 6a, b, from 28 to 90 days of curing, both the highest possible chloride migration coefficients and the differences between the average highest and the lowest possible migration coefficients decrease significantly. From 90 to 180 days of curing, changes in the average highest and the lowest possible migration coefficients in these two samples (M-NC-0 and M-NC-1) are almost negligible. Comparing M-NC-0 and M-NC-1, the CLDH-modified samples cover a lower range of possible chloride migration coefficient values at all ages. For sodium silicate-activated samples, the differences between the average highest and the lowest possible migration coefficients stayed relatively unchanged, while the highest possible chloride migration coefficients decrease slightly up to 180 days of curing.

The differences between the average highest and the lowest possible migration coefficients represent the range of values that the chloride migration coefficient could possibly fall into, and a smaller difference indicates a narrower range of within-sample variability. The results in Fig. 6 suggest that sodium carbonate-activated mortars without CLDH (M-NC-0) have significantly higher chloride migration coefficients than sodium silicate activated slag, even at extended curing times. While the CLDH modified sodium carbonate-activated slag mortar (M-NC-1) has higher *D*_nssm_ values than M-NS-0 up to 90 days of curing, the possible *D*_nssm_ ranges are more similar at 180 days of curing.

The non-steady state migration coefficients *D*_nssm_ are determined in part by the material microstructure, where a decrease in the critical pore size, as well as total intrudable porosity (as determined by MIP), could result in lower mobility of chlorides through the samples [36, 64]. The mobility of ionic species in gel pores (< 10 nm) is much slower than that in mesopores/macropores, and often considered insignificant for influencing the permeability of gas molecules and ionic species through the samples [61, 65]. A higher percentage of permeable pores could result in higher chloride transport parameters [66]. However, considering the short duration of this accelerated chloride test, the chemical interactions between chlorides and the gel binders would mostly take place locally in permeable pores, as it takes much longer for ionic species to move into the gel pores [67, 68].

Considering both the chloride binding capacities (Fig. 1) and MIP results (Table 2) shown in previous sections of this paper, it seems that the higher chloride migration coefficient of sample M-NC-0 compared with M-NS-0, even at 180 days of curing, is most likely caused by a combined effect of higher permeable porosity and lower chloride binding capacity. As for CLDH-modified sodium carbonate-activated mortar (M-NC-1-180d), even though it has lower permeable porosity than M-NS-0-180d, its stronger capacity to bind free chloride (compared with M-NS-0-180d) might be the reason that similar chloride coefficients have been observed for these two samples at 180 days of curing. Between these two factors, chloride binding capacity and the permeable porosity of the binders, it seems that the latter might be the dominant factor that controls the chloride migration coefficient under the currently tested methods. However, the NordTest method NT 492 involves non-steady state conditions and a short test duration (up to 48 h), while the chloride binding capacities were measured after 2 months of reaction, at equilibrium. The effect of chloride binding could therefore be underestimated in such an accelerated test method.

## Conclusions

Sodium carbonate-activated slag paste has a lower chloride binding capacity than with sodium silicate-activated slag paste. However, the incorporation of 5 wt% CLDH in the sodium carbonate-activated slag leads to an increase in chloride binding capacity by up to 120%, resulting in higher binding capacities than those of sodium silicate-activated slag pastes under the same testing conditions.

Sodium carbonate-activated samples have higher total intrudable porosity (by MIP), lower compressive strength, and higher chloride migration coefficients, compared with sodium silicate-activated slag materials at equivalent ages. However, with the incorporation of 5 wt% CLDH into sodium carbonate-activated slag mortar, the overall intrudable porosity of the sample has been effectively reduced, promoting a more refined pore structure. The effect of CLDH on densifying the microstructure, as well as increasing the chloride binding capacity of sodium carbonate-activated slag cement, results in higher compressive strength and a lower chloride migration coefficient, the values of which are almost comparable to those of sodium silicate-activated slag mortars produced under similar activation conditions. It is evident from the results obtained here that the addition of CLDH plays a critical role in improving the durability performance of sodium carbonate-activated slag cement.
